# Advanced hybrid closed-loop therapy in an older adult with 55-year type 1 diabetes: a CARE-based case report

**DOI:** 10.3389/fcdhc.2026.1870978

**Published:** 2026-06-26

**Authors:** Kamila Susuł, Bartłomiej Matejko, Paulina Suduł, Maciej T. Małecki, Tomasz Klupa, Katarzyna Cyranka

**Affiliations:** 1 Department of Metabolic Diseases and Diabetology, University Hospital in Kraków, Kraków, Poland; 2 Department of Metabolic Diseases, Centre for Advanced Technologies in Diabetes, Jagiellonian University Medical College, Kraków, Poland; 3 Department of Metabolic Diseases, Psychodiabetology Unit, Jagiellonian University Medical College, Kraków, Poland

**Keywords:** advanced hybrid closed-loop system, advanced hybrid closed loop (AHCL), continuous glucose monitoring (CGM), type 1 diabetes, diabetes distress, fear of hypoglycemia, insulin pump (CSII: continuous subcutaneous insulin infusion), MiniMed 780G system

## Abstract

This case describes a 66-year-old man with long-standing type 1 diabetes (diagnosed in 1969) complicated by polyneuropathy, autonomic neuropathy with hypoglycemia unawareness, and advanced diabetic retinopathy. Despite many years of treatment and consistent self-management, he experienced substantial glycemic variability, chronic hyperglycemia (HbA1c 9.2%), and a pronounced fear of hypoglycemia that led to insulin underdosing and maladaptive coping behaviors. Following admission to the Diabetes Clinic of the University Hospital in Krakow, structured diabetes education and psychological support were provided. The patient was subsequently enrolled in a research program and transitioned to the MiniMed™ 780G Advanced Hybrid Closed Loop (AHCL) system, which automatically adjusts insulin delivery based on continuous glucose monitoring. After nine months of AHCL use, the patient showed clinically meaningful improvement in CGM-derived metabolic outcomes (mean glucose 128 mg/dL, GMI 6.4%, TIR 91%, TBR 1%), with no episodes of severe hypoglycemia reported during follow-up. Standardized psychological assessment showed improvement in well-being and reductions in diabetes-related distress and depressive symptoms: WHO-5 increased from 9 to 14, DDS global score decreased from 3.2 to 1.8, PHQ-9 decreased from 7 to 3, and PAID global score decreased from 42 to 35. This CARE-based case report illustrates that AHCL therapy, when implemented together with structured education and psychological support, may be associated with improved glycemic outcomes and reduced psychological burden in selected older adults with long-standing type 1 diabetes. The findings should be interpreted cautiously because they derive from a single case.

## 1 Patient information and clinical context

A 66-year-old man with type 1 diabetes mellitus (T1DM), diagnosed in 1969, was referred to the Diabetes Clinic of the University Hospital in Kraków from a Regional Diabetes Clinic due to persistent metabolic imbalance and multiple chronic diabetes-related complications. His medical history included diabetic peripheral polyneuropathy, autonomic neuropathy manifested as hypoglycemia unawareness (lack of physiological symptoms signaling low blood glucose), and advanced diabetic retinopathy. Ophthalmologic interventions comprised bilateral retinal laser photocoagulation, posterior capsulotomy, and vitrectomy of both eyes following recurrent vitreous hemorrhages.

The patient reported a long history of difficulty maintaining stable glycemic control despite consistent self-care efforts. He described frustration, reduced confidence, and fatigue related to unpredictable glucose fluctuations. Repeated severe hypoglycemic episodes contributed to fear of hypoglycemia and avoidant behaviors, including intentional insulin underdosing. These factors affected quality of life and treatment decisions, supporting a biopsychosocial approach to management.

## 2 Diabetes management history and diagnostic assessment

Before admission to the University Hospital, the patient was managed with intensive insulin therapy using multiple daily injections (MDI). His regimen included insulin lispro (6–8 units before each main meal) and insulin glargine (18 units administered at approximately 10 p.m.), resulting in a total daily dose of 36–42 units. Despite adherence to this schedule, his glycated hemoglobin (HbA1c) level remained elevated at 9.2% (77 mmol/mol), indicating long-term suboptimal glycemic control.

The patient reported significant glycemic instability, experiencing both frequent hyperglycemia and numerous episodes of hypoglycemia, including severe events with loss of consciousness. The recurrent hypoglycemic episodes contributed to pronounced fear of hypoglycemia (FoH), leading to maladaptive self-management behaviors such as intentional underestimation of insulin doses before meals and avoidance of corrective boluses. These strategies, though protective in the short term, perpetuated chronic hyperglycemia and metabolic imbalance.

During hypoglycemic events, the patient tended to respond impulsively and ineffectively- he consumed excessive amounts of simple carbohydrates or administered intramuscular glucagon, resulting in rebound hyperglycemia. Such behavioral patterns reflected both emotional dysregulation and a lack of confidence in his ability to control blood glucose levels safely.

**Figure 1:**
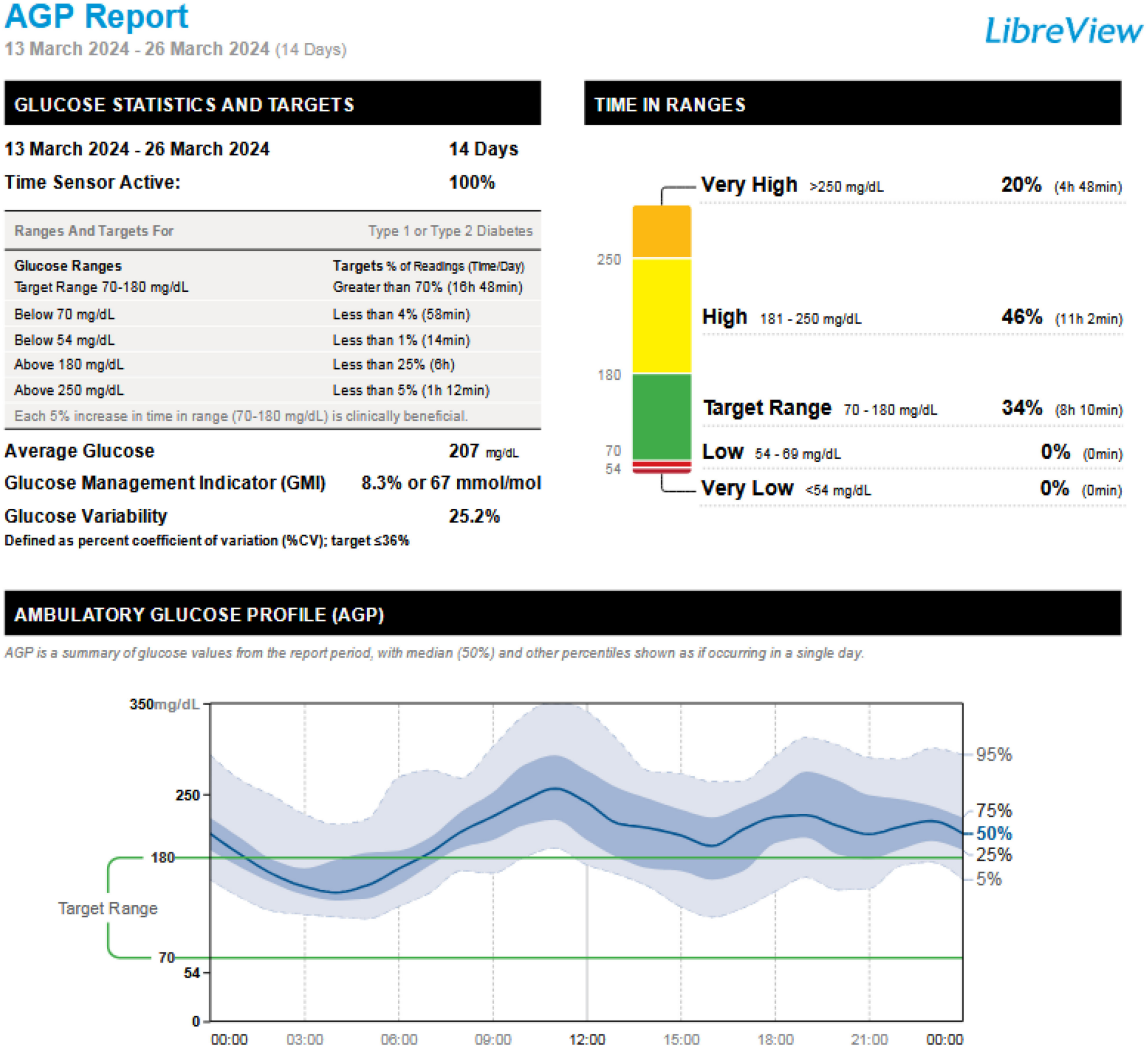
Ambulatory glucose profile (AGP) report from the FreeStyle Libre2 blood glucose monitoring system for the last 2 weeks prior to the visit to the Diabetes clinic.

Continuous glucose monitoring (CGM) data from the FreeStyle Libre2 system supported these observations. According to the Ambulatory Glucose Profile (AGP) from the two weeks preceding the hospital visit (**Figure 1**), mean glucose was 207 mg/dL, glucose management index (GMI) was 8.3%, and glucose variability (CV) was 25.2%. Time in Range (TIR, 70–180 mg/dL) was 34%, Time Above Range (TAR) was 46% for 180–250 mg/dL and 20% for >250 mg/dL. Time Below Range (TBR) was 0%. In this clinical context, baseline TBR of 0% was not interpreted as optimal glycemic safety, but rather as reflecting defensive hyperglycemia related to hypoglycemia unawareness, severe fear of hypoglycemia, intentional insulin underdosing, avoidance of corrective boluses, and preventive carbohydrate intake.

Overall, the pre-intervention pattern suggested a cycle of fear of hypoglycemia, avoidance of insulin correction, and persistent hyperglycemia. This interpretation was clinically relevant because the apparent absence of sensor-recorded hypoglycemia coexisted with a history of severe hypoglycemic events and hypoglycemia unawareness.

## 3 Therapeutic intervention

Upon admission to the Diabetes Clinic of the University Hospital in Kraków, therapeutic management began with a comprehensive re-education program. The patient received structured instruction on the principles of modern insulin therapy, appropriate recognition and management of hypoglycemia, and strategies for addressing persistent hyperglycemia. Additionally, he completed a refresher course on nutritional self-management and carbohydrate counting (1, 2).

Given his pronounced fear of hypoglycemia and its significant impact on self-care behaviors, the patient was referred for psychological consultation at the Psychodiabetology Clinic operating within the same institution. Psychological interventions focused on reducing anxiety, rebuilding self-efficacy, and restoring a sense of control over diabetes management. Cognitive-behavioral techniques and psychoeducation helped the patient identify maladaptive patterns—such as avoidance of insulin corrections and overcompensation after hypoglycemia—and replace them with safer, evidence-based strategies (3).

This integrative approach, combining medical and psychological care, was associated with improved understanding of diabetes self-management and greater readiness to engage with treatment. However, despite education and psychological support, glycemic targets were not fully achieved before transition to AHCL therapy.

The patient subsequently qualified for participation in a research project conducted by the Center for Advanced Diabetes Technologies at the Jagiellonian University Medical College, entitled: “Changing the treatment method for patients with type 1 diabetes (T1D) over the age of 65 to treatment with the MiniMed™ 780G Advanced Hybrid Closed Loop (AHCL) system: impact on metabolic control, quality of life, physical, cognitive, and vascular parameters.” (ClinicalTrials.gov reg. no. NCT06207838, protocol identifier 1072.61201.5.2023).

He was randomized to the intervention group, and his insulin therapy regimen was changed from multiple daily injections (MDI) to continuous subcutaneous insulin infusion using the MM780G system. The patient used the Guardian G4 sensor. The transition included individualized training in pump operation, data interpretation, and problem-solving in real-life situations. The patient was initiated on MiniMed™ 780G therapy initially in manual mode prior to activation of the AHCL algorithm. Initial pump settings included basal rates of 0.8 U/h between 00:00–03:00, 1.0 U/h between 03:00–09:00, and 0.9 U/h between 09:00–24:00. The active insulin time (AIT) was initially set at 3 hours. The carbohydrate-to-insulin ratios (ICR) were established at 10 g/U during nighttime hours (00:00–06:00) and 6 g/U during daytime hours (06:00–24:00). The insulin sensitivity factor (ISF) ranged from 60 mg/dL/U overnight to 45–50 mg/dL/U during daytime periods. Due to severe fear of hypoglycemia and impaired hypoglycemia awareness, the initial glucose target was set at 110 mg/dL during the first month of therapy. Following adaptation to the AHCL system and improvement in the patient’s sense of safety and treatment confidence, the glucose target was subsequently lowered to 100 mg/dL, and the active insulin time in automatic mode was shortened to 2 hours.

Prior to AHCL therapy, the patient did not calculate the exact amount of consumed carbohydrates and administered relatively fixed insulin doses with only minimal dose adjustments. Meal boluses were consistently underestimated in relation to carbohydrate intake, which contributed to suboptimal glycemic control. Prior to and during AHCL initiation, the patient participated in structured educational sessions with a dietitian focused on carbohydrate counting, meal bolus optimization, and nutritional management during insulin pump therapy. This educational support contributed to improved accuracy of carbohydrate estimation and reduced reliance on automated correction boluses during daily diabetes management.

Regular monthly follow-up visits provided ongoing medical and psychological support, facilitating adaptation to the new technology and reinforcing positive behavioral changes (4–7).

## 4 Intervention rationale: advanced hybrid closed-loop system

The MiniMed 780G system is an advanced hybrid closed-loop (AHCL) system that integrates.

CGM with automated insulin delivery. In the present case, the system was selected because the patient had persistent hyperglycemia, fear of hypoglycemia, hypoglycemia unawareness, and difficulty using corrective insulin safely during MDI therapy.

The system automatically adjusts basal insulin delivery and may deliver automated correction boluses based on sensor glucose trends. These functions were clinically relevant because they offered additional protection against both sustained hyperglycemia and anticipated hypoglycemia.

During transition, the patient received individualized training in pump use, meal bolusing, carbohydrate counting, data interpretation, and management of alarms and glucose excursions. Monthly follow-up visits were used to review CareLink data, reinforce education, and support psychological adaptation to the technology.

The therapeutic aim was to improve TIR, reduce sustained hyperglycemia and glycemic variability, maintain minimal hypoglycemia exposure, and reduce diabetes-related distress associated with constant self-management.

The psychological interpretation of AHCL use in this case is presented cautiously. The technology was not treated as a stand-alone solution; rather, it was implemented together with structured education and psychological support, which likely facilitated adherence, confidence, and safe use.

## 5 Follow-up and outcomes

After nine months of AHCL therapy, Medtronic CareLink data showed clinically meaningful improvement in metabolic outcomes and sustained device use.

**Figure 2:**
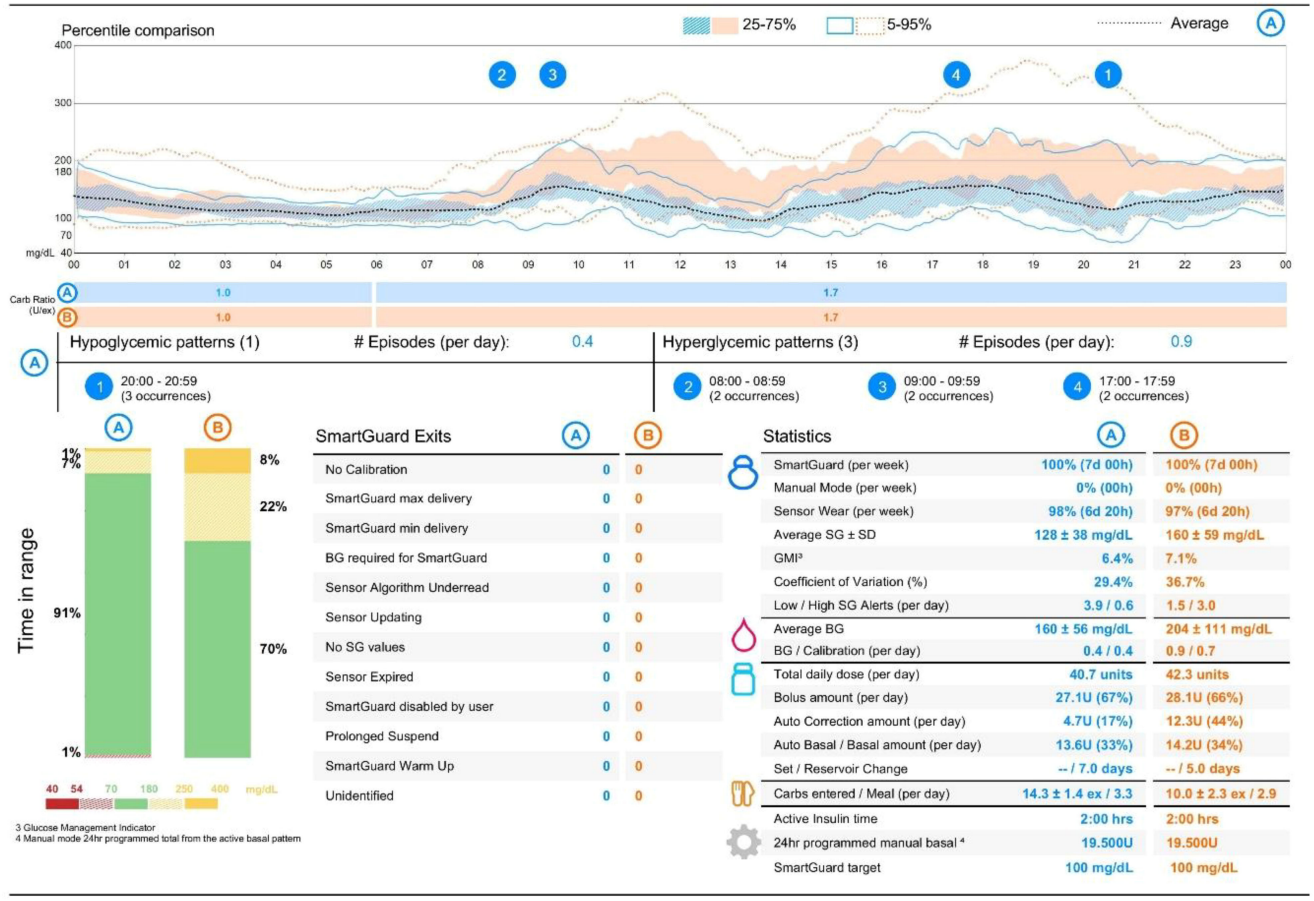
Assessment and progress report from Medtronic CareLink™ software.

As shown in **Figure 2**, the insulin pump operated in SmartGuard™ mode 100% of the time, indicating full system automation and consistent adherence to technology use. The upper panel of the report illustrates continuous glucose sensor readings over a 24-hour period (from midnight to midnight). Two datasets were compared: Data A (blue) — representing the last two weeks of active device use, and Data B (orange) — representing data collected during the third month of therapy.

During the most recent two-week period, mean glucose was 128 +/- 38 mg/dL and GMI was 6.4%. CV was 29.6%, remaining within the recommended range (<36%). Follow-up laboratory HbA1c was not available at the time of analysis; therefore, follow-up glycemic status was assessed using CGM-derived metrics, including GMI, together with available clinical follow-up data.

The total daily insulin dose (TDD) was 40.6 units. Basal insulin accounted for 33% (13.5 units), while boluses accounted for 67% (27.1 units), including 4.6 units (17%) delivered as automated correction boluses. The relatively low proportion of automated correction boluses may indirectly suggest improved meal bolus accuracy and more effective carbohydrate estimation after transition from MDI to pump therapy; however, carbohydrate-counting accuracy was not directly measured and this interpretation should therefore remain cautious.

The patient achieved the following CGM metrics:

- TIR (Time in Range 70–180 mg/dL): 91%.
- TAR (Time Above Range 180–250 mg/dL): 7%.
- TAR (Time Above Range >250 mg/dL): 1%.
- TBR (Time Below Range <70 mg/dL): 1%.

These results indicate favorable glycemic outcomes with low exposure to hypoglycemia. No episodes of severe hypoglycemia occurred during this period of insulin pump therapy.

The metabolic and psychological outcomes are summarized in **Table 1**, and the clinical timeline is presented in **Table 2**.

**Table 1:** Metabolic and psychological outcomes at baseline and follow-up.

Outcome	Baseline	Follow-up
HbA1c	9.2% (77 mmol/mol)	Not available
Mean sensor glucose	207 mg/dL	128 +/- 38 mg/dL
GMI	8.3%	6.4%
TIR 70–180 mg/dL	34%	91%
TBR <70 mg/dL	0%*	1%
TAR 180–250 mg/dL	46%	7%
TAR >250 mg/dL	20%	1%
Severe hypoglycemia	History of severe episodes with loss of consciousness	No episodes requiring third-party assistance were reported during follow-up
WHO-5	9	14
DDS	3.2 (global score)	1.8 (global score)
PHQ-9	7	3
PAID	42 (global score)	35 (global score)

*Baseline TBR 0% reflected defensive hyperglycemia and hypoglycemia avoidance rather than optimal glycemic safety.

**Table 2:** CARE-based clinical timeline.

Time point	Clinical event/finding
1969	Diagnosis of type 1 diabetes.
Pre-referral period	MDI therapy with insulin lispro before meals and insulin glargine at night; recurrent severe hypoglycemia and persistent hyperglycemia.
March 2024	FreeStyle Libre2 AGP: mean glucose 207 mg/dL, GMI 8.3%, TIR 34%, TBR 0% in the context of defensive hyperglycemia.
Diabetes Clinic admission	Structured re-education, carbohydrate-counting refresher, hypoglycemia-management education, and psychological consultation.
Research-program enrollment	Randomization to AHCL intervention and transition from MDI to MiniMed 780G with Guardian G4 sensor.
Months 1-9	Monthly medical and psychological follow-up with CareLink data review and ongoing education.
Month 9	Mean glucose 128 +/- 38 mg/dL, GMI 6.4%, TIR 91%, TBR 1%; no severe hypoglycemia reported; questionnaire scores changed in a direction consistent with improved well-being and lower psychological burden (WHO-5–9 to 14, DDS global score 3.2 to 1.8, PHQ-9–7 to 3, PAID global score 42 to 35).

From a psychological perspective, the patient reported reduced fear of hypoglycemia, greater perceived safety, and less need for constant vigilance. Standardized questionnaire results were consistent with this direction of change: WHO-5 increased from 9 to 14, DDS global score decreased from 3.2 to 1.8, PHQ-9 decreased from 7 to 3, and PAID global score decreased from 42 to 35. These findings suggest improved well-being and lower diabetes-related distress, depressive symptoms, and perceived diabetes burden; however, causality cannot be inferred from a single case report.

Overall, the combination of AHCL therapy, structured education, and psychological support was associated with clinically meaningful improvement in glycemic metrics and patient-reported psychological burden. Because this is a single case report, the findings should be interpreted as illustrative and hypothesis-generating rather than causal or generalizable evidence.

## 6 Discussion and conclusion

After nine months of AHCL therapy, the patient achieved guideline-consistent CGM targets with low exposure to hypoglycemia. This improvement occurred in the context of structured education, regular follow-up, and psychological support. No episodes of severe hypoglycemia requiring third-party assistance were reported during the follow-up period.

At transition, the patient was 66 years old and had lived with type 1 diabetes for 55 years. He had advanced microvascular complications, hypoglycemia unawareness, and long-standing fear of hypoglycemia. These features make the case clinically relevant, but they also limit generalizability to broader older adult populations with type 1 diabetes.

The introduction of AHCL therapy was associated with improved glucose metrics and greater perceived safety (8–10). The baseline TBR of 0% was interpreted in context: it likely reflected defensive hyperglycemia and intentional insulin underdosing rather than optimal protection from hypoglycemia.

From a clinical standpoint, this case suggests that AHCL therapy can be feasible in selected older adults with long-standing type 1 diabetes when accompanied by structured education and close follow-up. However, the single-case design does not allow causal inference or estimation of treatment effects.

From a psychological standpoint, the case highlights the importance of assessing emotional barriers such as fear of hypoglycemia, diabetes distress, and loss of confidence in self-management. In this patient, questionnaire scores changed in a direction consistent with improved well-being and reduced psychological burden (WHO-5–9 to 14; DDS global score 3.2 to 1.8; PHQ-9–7 to 3; PAID global score 42 to 35). These findings should be interpreted descriptively and cautiously, given the single-case design and the concurrent use of AHCL therapy, structured education, and psychological support.

In conclusion, chronological age alone should not exclude patients from consideration for advanced diabetes technologies. In selected older adults, AHCL therapy combined with education and psychological support may be associated with improved glycemic outcomes and reduced perceived diabetes burden. Further studies are needed to determine the generalizability of these observations.

## Data Availability

The raw data supporting the conclusions of this article will be made available by the authors, without undue reservation.
